# Investigating the neural ensembles underlying sundowning in an Alzheimer’s disease mouse model

**DOI:** 10.1002/alz.089605

**Published:** 2025-01-03

**Authors:** Michelle Jin, Holly C Hunsberger, Alicia R Whye, Simon Ogundare, Nicole Alvarado, Sophia Sorid, Marcos Lanio, Christine Ann Denny

**Affiliations:** ^1^ Columbia Irving Medical Center, New York, NY USA; ^2^ Rosalind Franklin University, Chicago, IL USA; ^3^ Weill Cornell Medicine, New York, NY USA; ^4^ Columbia University, New York, NY USA; ^5^ Barnard University, New York, NY USA; ^6^ Stony Brook, Ronkonkoma, NY USA; ^7^ Research Foundation for Mental Hygiene, New York, NY USA

## Abstract

**Background:**

As high as 50% of Alzheimer’s disease (AD) patients experience “sundowning”, which refers to an increased severity of neuropsychiatric symptoms (NPS), including agitation, confusion, and anxiety, selectively in the evening. Although sundowning significantly influences the decision to institutionalize patients, few preclinical models of this phenomenon exist and the underlying neural mechanisms are unknown. Here, we establish a model of sundowning by phenotyping the sleep‐wake cycle and anxiety and exploratory behavior at different times of day in an AD mouse model. We then investigate differences in brain‐wide activity patterns present at sundown and sunrise time in AD and control (Ctrl) mice.

**Method:**

We recorded sleep‐wake activity in control (Ctrl) and APP/PS1de9 (AD) mice across 2‐, 6‐, 12‐, and 24‐month‐old male and female mice. Next, we measured anxiety/locomotion in 2‐, 12‐, and 24‐month‐old Ctrl and AD mice during the murine evening (ZT22‐0, sundown) and murine morning (ZT12‐14, sunrise) using the elevated‐plus maze (EPM) and open field (OF) assays. To identify brain‐wide ensemble patterns mediating sundowning, we bred the ArcCreER^T2^ x EYFP activity‐dependent tagging strategy with the AD line, which allows for temporally precise labelling of two active neural populations. We then applied a custom pipeline to label and map ensembles active during exposure to the EPM at sundown and sunrise.

**Result:**

We found a degradation in strength of sleep‐wake rhythms with aging selectively in AD mice. We further found that older AD mice (24‐month‐old males and 12‐ and 24‐month‐old females) preferentially explore the open arms of the EPM and display hyperlocomotion in the OF (24‐month‐old males and 24‐month‐old females) selectively during sundown. Network analysis of mapping results showed selective decreases in topological measures of connectivity, such as degree, clustering, and efficiency, in AD mice at sundown but not sunrise time when compared with Ctrl mice.

**Conclusion:**

We identified a striking behavioral abnormality in an AD mouse model resembling “nighttime wandering” and agitation seen in sundowning patients. Mapping of concurrent brain‐wide activity patterns showed global decreases in network connectivity at sundown time in contrast to sunrise time in AD mice, revealing novel insights about the neural patterns underlying this debilitating syndrome.

